# Association between Right Ventricular Function and Exercise Capacity in Patients with Chronic Heart Failure

**DOI:** 10.3390/jcm11041066

**Published:** 2022-02-18

**Authors:** Kazumasa Ohara, Teruhiko Imamura, Hiroyuki Ihori, Kenichi Chatani, Makoto Nonomura, Tomoki Kameyama, Hiroshi Inoue

**Affiliations:** 1 Internal Medicine, Saiseikai Toyama Hospital, Toyama 931-8533, Japan; k.ohara175@gmail.com (K.O.); pre0890@gmail.com (H.I.); chatani.kenichi@gmail.com (K.C.); m-nonomura@saiseikai-toyama.jp (M.N.); tkameyam@gmail.com (T.K.); h-inoue@saiseikai-toyama.jp (H.I.); 2 The Second Department of Internal Medicine, University of Toyama, Toyama 930-0194, Japan

**Keywords:** peak oxygen consumption, TAPSE, right ventricular failure

## Abstract

Background: The association between right ventricular function and exercise capacity in patients with chronic heart failure remains uncertain. Several studies very recently mentioned the association between right ventricular reserve and exercise capacity, whereas the implication of tricuspid annular plane systolic excursion (TAPSE) remains uninvestigated. We aimed to assess the impact of TAPSE on exercise capacity in cardiac rehabilitation candidates. Methods: Data from patients with chronic heart failure who received cardiopulmonary exercise tests and transthoracic echocardiography prior to cardiac rehabilitation were retrospectively collected, and their association was investigated. Results: A total of 169 patients with chronic heart failure (70.3 ± 11.7 years old, 74.6% men) were included. Tertiled tricuspid annular plane systolic excursion significantly stratified anaerobic threshold (10.2 ± 2.2, 11.4 ± 2.2, and 12.2 ± 2.8 mm; *p* < 0.01) and peak oxygen consumption (15.9 ± 4.5, 18.3 ± 5.3, and 19.8 ± 5.6 mm; *p* < 0.01). In the multivariate logistic regression analyses, TAPSE was an independent factor associated with anaerobic threshold and peak oxygen consumption (*p* < 0.05 for both). Conclusions: Right ventricular impairment was associated with reduced exercise capacity in patients with chronic heart failure. Such knowledge would be useful to estimate patients’ exercise capacity and prescribe cardiac rehabilitation. Its longitudinal association and clinical implication need further studies.

## 1. Background

Exercise capacity has been a matter of great concern thus far given that it is considerably associated with mortality and morbidity in patients with heart failure. Exercise capacity is predominantly affected by the amount of skeletal muscle in patients with heart failure, whereas the impact of cardiac function on exercise capacity might be partial [1].

Nevertheless, impaired right heart function was recently demonstrated to be associated with reduced exercise capacity in patients with coronary artery disease [2], whereas little is known about the association between right ventricular function and exercise capacity in patients with chronic heart failure. The clarification of these associations would improve prognostic stratification and therapeutic strategy for those with impaired right heart function. 

Theoretically, systolic right heart failure would decrease forward flow toward pulmonary vasculature and impairs oxygen exchange. Diastolic right heart failure would exacerbate systemic congestion and impair peripheral circulation. Both of these would worsen exercise capacity. A further concern is whether cardiac rehabilitation can improve exercise capacity, particularly for those with impaired right ventricular function.

Very recently, the association between right ventricular function/reserve and exercise capacity has been investigated among various types of cohorts, including hospitalized heart failure patients, systolic heart failure patients, and diastolic heart failure patients [3,4,5,6]. The association among candidates for cardiac rehabilitation remains uncertain. 

In this study, we investigated the association between right ventricular function assessed using echocardiography and exercise capacity quantified by cardiopulmonary exercise tests in patients with chronic heart failure prior to cardiac rehabilitation. We also investigated the impact of baseline right ventricular function on the effect of cardiac rehabilitation. 

## 2. Methods

### 2.1. Patient Selection

Consecutive patients with chronic heart failure who received cardiac rehabilitation between 2015 and 2020 at our institute were considered for inclusion in this retrospective study. Patients with active angina pectoris, those with a history of acute coronary syndrome within the past 3 months, those with unstable hemodynamics, those with symptomatic congestion, those with critical arrhythmia, those with active systemic infection, or those unable to participate in cardiac rehabilitation due to various other comorbidities did not receive cardiac rehabilitation and were excluded from this study. The diagnosis of heart failure was made according to the Framingham criteria. Patients who received cardiopulmonary exercise tests and transthoracic echocardiography within one week before cardiac rehabilitation were finally included for the present analysis. The institutional review board approved the study protocol. All participants gave written informed consent beforehand.

### 2.2. Cardiopulmonary Exercise Test

A symptom-limited cardiopulmonary exercise test was performed using a bicycle ergometer with a ventilator and expired gas analyzer (Minato Medical Science, Osaka, Japan), according to the American Heart Association’s guidelines [7]. All patients initiated the tests at 20 W for a 4 min warm-up period following the initial 3 min rest period and underwent a 10 W per minute ramp incremental protocol. 

Continuous data of oxygen consumption (VO2), production of carbon dioxide (VCO2), and minute ventilation (VE) were measured during the tests on a breath-by-breath basis. Peak VO2 (PVO2) was defined as the highest VO2 noted during the exercise. Anaerobic threshold (AT) was determined using the V-slope, ventilator equivalents, and end-tidal pressure methods by the attending experts [8]. 

Considering the Borg scale, which indicates patients’ objective fatigue in the range of 6–20, a score above 17 was targeted to terminate the tests. The respiratory exchange ratio was targeted at above 1.10.

### 2.3. Transthoracic Echocardiography

Transthoracic echocardiography was performed according to the American Society of Echocardiography guidelines [9]. Right ventricular end-diastolic and systolic areas were traced from the apical four-chamber right ventricle-focused view, and right ventricular fractional area change (RVFAC) was calculated. An M-mode cursor was oriented at the junction of the tricuspid valve plane and the right ventricular free wall to measure tricuspid annular plane systolic excursion (TAPSE). 

The peak early diastolic transmitral flow velocity (E) was measured by pulse wave Doppler echocardiography. Tissue Doppler measurement of mitral annular velocity (e’) at the left ventricular lateral wall was also measured. E/e’ was calculated as an index of diastolic function. LV end-diastolic diameter (LVDd) was measured through parasternal long-axis views at mitral tip level. Left ventricular ejection fraction was calculated using the modified Simpson’s method using 2- and 4-chamber views or using the Teichholz method from the M-mode image.

### 2.4. Cardiac Rehabilitation

Cardiac rehabilitation was performed in a standard manner according to the Japanese Circulation Society guidelines [10]. For exercise intensity, the anaerobic metabolism threshold was measured from a cardiopulmonary exercise test, and the heart rate or watt intensity 1 min before the anaerobic metabolism threshold was used. Patients continued 1–3 weekly bicycle ergometer supervised aerobic exercises and low-intensity resistance training (hip abduction, hip extension, half squat, and calf raise) for 10–20 min per session as well as 1–2 weekly unsupervised exercises with walking for five months. Heart failure education for self-care and behavioral strategies were carried out.

### 2.5. Statistical Analysis

Continuous variables were expressed as the mean and standard deviation. Categorical variables were expressed as numbers and percentages. The association between clinical parameters including TAPSE and exercise capacity data including AT and PVO2 were investigated using linear regression analyses. Variables significant in the univariable analysis were included in the multivariable analysis with the forced entry method. Variables tertiled by TAPSE were compared using one-way analysis of variance and multiple comparisons with Tukey methods. Among patients who received 5-month cardiac rehabilitation, changes in AT and PVO2 following cardiac rehabilitation were assessed in those with normal TAPSE (>16 mm, which was a lower limit of the normal range) and in those with abnormally low TAPSE ≤ 16 mm separately using paired *t*-tests. A value of *p* < 0.05 was considered statistically significant. Statistical analyses were performed using JMP^®^ 12 (SAS Institute Inc., Cary, NC, USA).

## 3. Results

### 3.1. Baseline Characteristics

**Figure 1:**
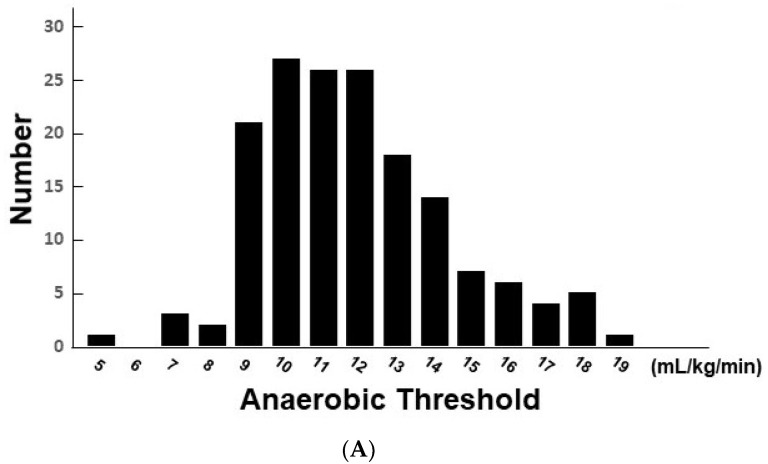

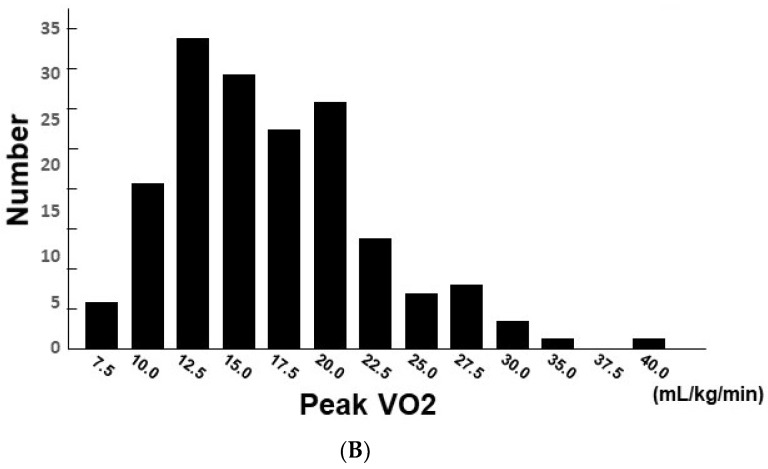
Distributions of AT (**A**) and PVO2 (**B**).

Among 345 patients, a total of 169 patients with chronic heart failure (70.3 ± 11.7 years old, 74.6% men) were finally included (Table 1). Most of the patients had New York Heart Association functional class II. Left ventricular end-diastolic diameter was 51.0 ± 8.7 mm and left ventricular ejection fraction was 52.3 ± 16.9%. The results of the cardiopulmonary exercise test are summarized in Table 2. AT and PVO2 were distributed widely (Figure 1A,B).

Baseline characteristics were tertiled by TAPSE in supplemental Table S1. Patients with the lowest TAPSE had more advanced heart failure due to non-ischemic etiology. 

### 3.2. Association between Clinical Parameters and AT

**Figure 2:**
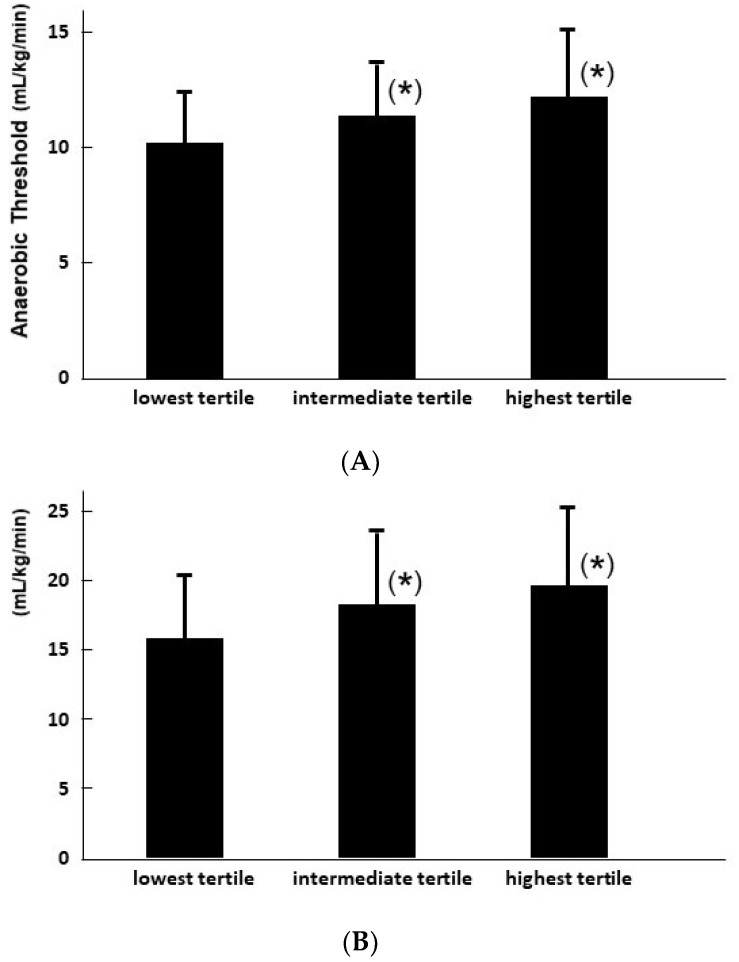
AT (**A**) and PVO2 (**B**) levels stratified by the tertiled TAPSE. * *p* < 0.05 compared with the lowest title by post-hoc Tukey’s test.

In the univariable analyses, several clinical variables including echocardiographic parameters were significantly associated with the AT level. TAPSE was independently associated with the AT level when adjusted for variables significant in the univariable analyses, as well as age and NYHA class (*p* < 0.05 for both; Table 3). Tertiled TAPSE significantly stratified the levels of AT (*p* < 0.01; Figure 2A).

### 3.3. Association between Clinical Parameters and PVO2

In the same manner, TAPSE was independently associated with PVO2 when adjusted for variables significant in the univariable analyses as well as age (*p* < 0.05 for all; Table 4). Tertiled TAPSE significantly stratified the levels of PVO2 (*p* < 0.01; Figure 2B). 

### 3.4. Comparison of Changes in AT and PVO2 Levels According to Initial TAPSE Levels

A total of 56 patients completed the five-month cardiac rehabilitation. All 56 patients attended >80% of the scheduled sessions. Of them, 35 had normal TAPSE above 16 mm at baseline and 21 had abnormally low TAPSE ≤ 16 mm at baseline. Patients with low TPASE had more advanced heart failure due to non-ischemic etiology (Supplementary Table S2). 

**Figure 3:**
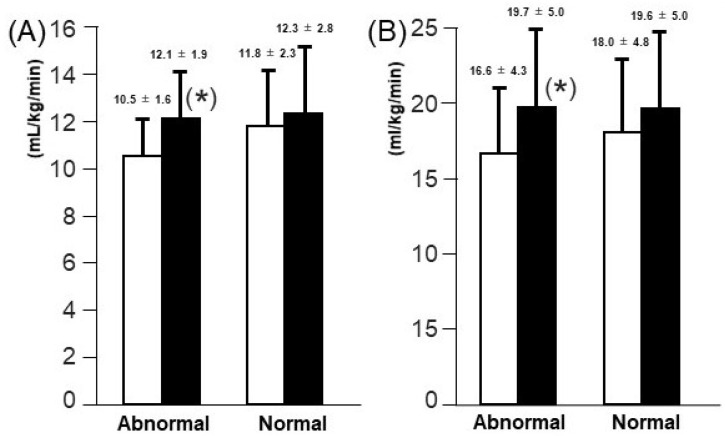
Changes in AT (**A**) and PVO2 (**B**) following the 5-month cardiac rehabilitation in the normal and abnormal TAPSE groups. * *p* < 0.05 compared to baseline. Open bars, baseline; solid bars, 5-month follow-up.

Following 5-month cardiac rehabilitation, both AT and PVO2 improved significantly (from 11.3 ± 2.2 to 12.2 ± 2.5 mL/min/kg for AT and from 17.5 ± 4.7 to 19.7 ± 4.9 mL/min/kg for PVO2, *p* < 0.01 for both). Both AT and PVO2 remained high following cardiac rehabilitation in patients with normal TAPSE (*p* > 0.05 for both; Figure 3). Interestingly, in patients with abnormally low TAPSE, AT and PVO2 increased significantly following cardiac rehabilitation (*p* < 0.05; Figure 3). 

## 4. Discussion

In this study, we investigated the association between right ventricular function quantified by TAPSE and exercise capacity quantified by AT and PVO2 in patients with chronic heart failure. The major findings are as follows. Firstly, impaired right ventricular function represented by lower TAPSE was independently associated with reduced exercise capacity indicated by lower AT and PVO2, respectively. Secondly, exercise capacity remained high in patients with preserved baseline TAPSE, whereas an improvement in exercise capacity was observed following 5-month cardiac rehabilitation in patients with low baseline TAPSE. 

### 4.1. Association between Right Ventricular Function and Exercise Capacity

Various factors are associated with exercise capacity in patients with chronic heart failure, but there is a scarcity of studies investigating the impact of right ventricular function on exercise capacity thus far [2,11]. Elevated left ventricular end-diastolic pressure and pulmonary hypertension, both of which are indirectly presented by low TAPSE, impair appropriate oxygen exchange during exercise and are dominant determinants of exercise capacity in the heart failure cohort. 

Incremental pulmonary artery pressure during exercise is a significant afterload on the right ventricle [5]. Impaired systolic failure of the right ventricle, indicated by low TAPSE, cannot propel the blood against the elevated afterload and supply appropriate deoxygenized blood to the lungs [4,6]. Low levels of TAPSE and systolic pulmonary artery pressure, both of which indicate impaired right ventricular systolic function, were associated with poor exercise ability [12], a phenomenon of right ventricle–pulmonary artery uncoupling [3]. 

A low TAPSE also indicates diastolic dysfunction of the right ventricle, accompanying systemic congestion. Akiyama and colleagues demonstrated a parallel improvement in systemic congestion and TAPSE despite unchanged left ventricular end-diastolic volume and cardiac output during hospitalization for acute heart failure [13]. Borlaug and colleagues demonstrated the association between impaired right ventricular diastolic function during exercise and incremental intra-ventricular filling pressure during exercise [6]. The existence of systemic congestion is in general associated with reduced skeletal muscle circulation and impaired exercise capacity. 

Ghio and colleagues reported that impaired right heart function was a prognostic risk factor independent of pulmonary hypertension in patients with chronic heart failure [14]. Other investigators demonstrated that exercise-induced right ventricular dysfunction was independently associated with poor clinical outcomes in patients with asymptomatic degenerative mitral regurgitation [15]. 

### 4.2. Impact of Cardiac Rehabilitation for Those with Impaired Right Ventricular Function

Fortunately, patients with low TAPSE, i.e., impaired right ventricular function, could also enjoy improvement in exercise capacity following cardiac rehabilitation, as shown in the present study. Cardiac rehabilitation has multidisciplinary effects in improving exercise capacity and prognosis in patients with chronic heart failure. Cardiac rehabilitation might have a direct effect in improving skeletal muscle and exercise capacity [16,17]. Improved respiratory muscle function through cardiac rehabilitation would improve pulmonary hypertension and reduce afterload on the right ventricle. Improvement in left ventricular function through cardiac rehabilitation, which was demonstrated by echocardiographic reverse remodeling, an increase in cardiac output during exercise, and a reduction in plasma B-type natriuretic peptide levels, would ameliorate pulmonary hypertension and thereby reduce afterload on the right ventricle [18]. 

Further studies are warranted to propose a specific protocol for cardiac rehabilitation for those with low TAPSE at baseline. Cardiac rehabilitation is essential also for those with normal TAPSE at baseline to maintain their exercise capacity, given the progressive nature of chronic heart failure.

### 4.3. Limitations

We cannot ignore that there are several limitations. First, this is a retrospective study using a moderate sample size cohort. Cardiac rehabilitation was not continued completely for all outpatients. We performed multivariable analyses, but any other uninvestigated confounders might exist. Second, we used TAPSE as a representative of right ventricular function. There are several other parameters associated with right ventricular function, including three-dimensional right ventricular ejection fraction and right ventricular strain analyses. However, TAPSE is easy to measure without any requirement of whole right heart images and expert techniques. Third, exercise-induced pulmonary hypertension is one of the dominant determinants of impaired exercise capacity. Incremental afterload on the right ventricle during exercise increases transverse contraction and reduces longitudinal shortening [19]. TAPSE is an ideal marker to assess longitudinal contractile function in the right ventricle [18]. This is the rationale for why we used TAPSE among several right-ventricle-related markers. Finally, we assessed the changes in exercise capacity following cardiac rehabilitation. Detailed prescriptions varied for each patient. Cardiac rehabilitation was prescribed appropriately according to each patient’s exercise capacity, cardiac function, daily life activity, and individual goals. 

## 5. Conclusions

Right ventricular impairment was associated with reduced exercise capacity in patients with chronic heart failure. This knowledge would be useful to estimate patients’ exercise capacity and prescribe cardiac rehabilitation. Its longitudinal association and clinical implications remain concerns for the future.

## Figures and Tables

**Table 1:** Baseline characteristics.

	Total (*n* = 169)
Demographics	
Men	126 (74.6%)
Age (years)	70.3 ± 11.7
Hypertension	133 (78.7%)
Diabetes mellitus	57 (33.7%)
Dyslipidemia	128 (75.7%)
Chronic kidney disease	64 (37.9%)
Anemia	62 (36.7%)
Current smoking	34 (20.1%)
New York Heart Association functional class	2.2 ± 0.5
Plasma B-type natriuretic peptide (pg/mL)	150.5 [54.0; 312.2]
Etiology	
Ischemic heart disease	118 (69.8%)
Valvular disease	12 (7.1%)
Cardiomyopathy	22 (13.0%)
Arrhythmia	4 (2.4%)
Congenital heart disease	2 (1.2%)
Others	11 (6.5%)
Echocardiography	
Left ventricular end-diastolic diameter (mm)	51.0 ± 8.7
Left ventricular ejection fraction (%)	52.3 ± 16.9
Tricuspid annular plane systolic excursion (mm)	18.3 ± 4.9
Transmitral E velocity (m/sec)	0.77 ± 0.24
E lateral e’ ratio	9.8 ± 3.9

Values are number of patients (%), mean ± SD or median [interquartile range]. E = early-diastolic; e’ = early-diastolic mitral annular velocity.

**Table 2:** Cardiopulmonary exercise test.

	Anaerobic Threshold Level	Peak Level
Heart rate (bpm)	94.3 ± 17.6	121.2 ± 27.9
Systolic blood pressure (mmHg)	143.6 ± 26.7	165.0 ± 34.5
VO2 (mL/kg/min)	11.3 ± 2.5	18.0 ± 5.7
VCO2 (mL/kg/min)	10.7 ± 2.6	21.2 ± 7.3
VE (L/min)	23.2 ± 5.5	49.0 ± 20.1

Values are mean ± SD. VO2 = oxygen uptake, VCO2 = carbon dioxide output, VE = ventilatory equivalent.

**Table 3:** Association between AT and clinical parameters including TAPSE.

	Univariate Analysis		Multivariate Analysis	
	Beta Value	*p* Value	Beta Value	*p* Value
Age	−0.9171	0.01 *	−0.0648	0.02
Hypertension	0.0928	0.23 *		
Diabetes mellitus	0.1881	0.01 *	0.3053	0.20
Dyslipidemia	0.0732	0.06		
Chronic kidney disease	0.2065	<0.01 *	0.2818	0.20
Anemia	0.1458	0.04 *	0.0877	0.72
Smoking	−0.0026	0.97		
New York Heart Association functional class	0.0534	<0.01 *	−10.698	0.03 *
Left ventricular end-diastolic diameter	0.0369	0.89		
Left ventricular ejection fraction	0.1015	0.85		
Tricuspid annular plane systolic excursion	0.6921	<0.01 *	0.1224	0.01 *
Transmitral E velocity	−0.0172	0.03	−0.8610	0.47
Lateral E/e’ ratio	−0.4069	<0.01 *	−0.0479	0.51
Plasma B-type natriuretic peptide	−21.048	0.27		

* *p* < 0.05. E = early-diastolic. e’ = early-diastolic mitral annular velocity.

**Table 4:** Association between PVO2 and clinical parameters including TAPSE.

	Univariate Analysis		Multivariate Analysis	
	Beta Value	*p* Value	Beta Value	*p* Value
Age	−10.985	<0.01 *	−0.196	<0.01 *
Hypertension	0.008	0.82		
Diabetes mellitus	0.108	<0.01 *	0.480	0.25
Dyslipidemia	0.041	0.21		
Chronic kidney disease	0.034	<0.01 *	0.719	0.06
Anemia	0.166	<0.01 *	0.656	0.14
Current smoking	−0.057	0.10		
New York Heart Association functional class	−0.017	0.01 *	−0.390	0.66
Left ventricular end-diastolic diameter	0.087	0.49		
Left ventricular ejection fraction	0.061	0.80		
Tricuspid annular plane systolic excursion	0.319	<0.01 *	0.231	<0.01 *
Transmitral E velocity	−0.014	<0.01 *	−2.331	0.28
Lateral E/e’ ratio	−0.257	<0.01 *	−0.073	0.57
Plasma B-type natriuretic peptide	−13.800	0.11		

* *p* < 0.05. E = early-diastolic. e’ = early-diastolic mitral annular velocity.

## Data Availability

Data are available upon reasonable request.

## Supplementary Materials

The following supporting information can be downloaded at: https://www.mdpi.com/article/10.3390/jcm11041066/s1, Table S1: Baseline characteristics according to tertile of TAPSE; Table S2: Baseline characteristics according to TAPSE.
