# Supplementary material for: Association between Right Ventricular Function and Exercise Capacity in Patients with Chronic Heart Failure
Source: J Clin Med. 2022 Feb 18;11(4):1066. doi: 10.3390/jcm11041066 (PMC8877496; doi:10.3390/jcm11041066)
Supplement: Supplementary file 1 [file jcm-11-01066-s001.zip › jcm-1587053-supplementary.pdf]

Supplementary Table S1. Baseline characteristics according to tertile of TAPSE.

	Lowest (N = 56)	Intermediate (N = 56)	Highest (N = 57)	p value
Demographics				
Men	42 (75%)	40 (71.4%)	44 (77.2%)	0.77
Age (years)	70.6 ± 12.3	69.3 ± 11.8	71.0 ± 11.0	0.72
Hypertension	44 (78.6%)	42 (75.0%)	47 (82.5%)	0.62
Diabetes mellitus	21 (37.5%)	21 (37.5%)	15 (26.3%)	0.34
Dyslipidemia	42 (75.0%)	43 (76.7%)	43 (75.4%)	0.97
Chronic kidney disease	23 (41.1%)	26 (46.4%)	15 (26.3%)	0.07
Anemia	21 (37.5%)	21 (37.5%)	20 (35.0%)	0.95
Current smoking	15 (26.8%)	11 (19.6%)	8 (14.0%)	0.24
NYHA functional class	2.4 ± 0.6	2.1 ± 0.4 *	2.1 ± 0.4 *	< 0.01
Plasma BNP (pg/mL)	235.4 [120.8; 582.4]	130.1 [50.5; 282.1] *	84.3 [43.1; 210.8] *	< 0.01
Etiology				< 0.03
Ischemic heart disease	25 (44.6%)	45 (80.4%)	48 (84.2%)	
Valvular disease	11 (19.7%)	1 (1.8%)	0 (0%)	
Cardiomyopathy	11 (19.7%)	7 (12.5%)	4 (7.0%)	
Arrhythmia	4 (7.1%)	0 (0%)	2 (3.5%)	
Congenital heart disease	1 (1.8%)	1 (1.8%)	0 (0%)	
Others	3 (5.4%)	2 (3.6%)	3 (5.3%)	
Echocardiography				
LVDd (mm)	50.9 ± 10.9	50.5 ± 8.2	51.4 ± 6.5	0.85
LVEF (%)	48.7 ± 18.1	54.1 ± 16.6 *	59.0 ± 13.6 *	< 0.01
TAPSE (mm)	12.8 ± 2.1	18.4 ± 1.3 *	23.6 ± 2.5 *	< 0.01
E velocity (m/sec)	0.83 ± 0.29	0.74 ± 0.22	0.73 ± 0.20	0.07
E lateral e' ratio	11.7 ± 4.8	9.1 ± 3.5 *	9.4 ± 3.2	0.03

Values are number of patients (%), mean ± SD or median [interquartile range]. * $p < 0.05$ compared to lowest TAPSE. NYHA = New York Heart Association; BNP = B-type natriuretic peptide. LVDd = Left ventricular end-diastolic diameter; LVEF = Left ventricular ejection fraction. TAPSE = Tricuspid annular plane systolic excursion. E = Transmitral early-diastolic; e' = early-diastolic mitral annular velocity.

Supplementary Table S2. Baseline characteristics according to TAPSE.

	**Low (N = 21)**	**Normal (N = 35)**	**P value**
Demographics			
Men	14 (66.7%)	24 (68.6%)	0.88
Age (years)	73.1 ± 9.8	74.7 ± 6.8	0.47
Hypertension	17 (81.0%)	27 (77.1%)	0.74
Diabetes mellitus	6 (28.6%)	10 (28.6%)	1.00
Dyslipidemia	17 (81.0%)	30 (85.7%)	0.64
Chronic kidney disease	9 (42.9%)	15 (42.9%)	1.00
Anemia	9 (42.9%)	13 (37.1%)	0.67
Current smoking	6 (28.6%)	4 (11.4%)	0.10
NYHA functional class	2.3 ± 0.5	2.0 ± 0.4	0.01 *
Plasma BNP (pg/mL)	149.6 (86.3, 404.7)	82.2 (46.3, 188.5)	0.06
Etiology			0.04 *
Ischemic heart disease	12 (57.1%)	30 (85.7%)	
Valvular disease	5 (23.8%)	1 (2.9%)	
Cardiomyopathy	2 (9.5%)	2 (5.7%)	
Arrhythmia	1 (4.8%)	0 (0%)	
Congenital heart disease	0 (0%)	0 (0%)	
Others	1 (4.8%)	2 (5.7%)	
Echocardiography			
LVDd (mm)	46.6 ± 11.5	49.5 ± 8.3	0.28
LVEF (%)	50.6 ± 14.7	59.2 ± 14.3	0.04 *
TAPSE (mm)	12.5 ± 2.2	21.4 ± 2.8	< 0.01 *
Transmitral E velocity (m/sec)	0.80 ± 0.27	0.70 ± 0.23	0.18
E lateral e' ratio	10.6 ± 5.5	9.1 ± 3.5	0.23

Values are number of patients (%), mean ± SD or median (interquartile range). * $p < 0.05$. NYHA = New York Heart Association; BNP = B-type natriuretic peptide; LVDd = Left ventricular end-diastolic diameter; LVEF = Left ventricular ejection fraction; TAPSE = Tricuspid annular plane systolic excursion; E = early-diastolic; e' = early-diastolic mitral annular velocity.
